# Weight management interventions for pregnant women from minority ethnic populations living in the United Kingdom: a scoping review

**DOI:** 10.3389/fpubh.2026.1885429

**Published:** 2026-09-25

**Authors:** Baraa Morsy, Britzer Paul Vincent, Sarmad Raza, Bhavneet Singh, Nasreen Ali

**Affiliations:** Faculty of Health and Social Sciences, University of Bedfordshire, Luton, United Kingdom

**Keywords:** disparity, inequalities (MeSH), intervention, minority ethnic (ME), pregnancy, scoping review, United Kingdom, weight management

## Abstract

**Background:**

Maternal obesity disproportionately affects women from minority ethnic backgrounds in the United Kingdom, who face substantially higher risks of gestational diabetes mellitus, hypertensive disorders, and maternal mortality than White British women. Despite well-documented disparities and relevant National Institute for Health and Care Excellence guidance, the evidence base for culturally appropriate weight management during pregnancy remains poorly mapped.

**Aim:**

To map and synthesize available evidence on weight management interventions during pregnancy among minority ethnic women in UK settings.

**Methods:**

A scoping review was conducted following the Arksey and O'Malley (2005) framework and reported according to PRISMA-ScR. Seven electronic databases were searched from 2010 to 2025. Eligible studies assessed weight management interventions among pregnant minority ethnic women in the UK, or multi-ethnic UK cohorts with ethnicity-disaggregated data.

**Findings:**

Three studies were included with 1,741 participants, published between 2016 and 2025. Three domains were identified: multi-component behavioral interventions applied without cultural adaptation; differential engagement by ethnicity; and limited ethnicity-stratified reporting of effectiveness, with exploratory subgroup analyses that were underpowered and inconclusive. The reviewed studies demonstrated limited cultural adaptation and inadequate ethnicity-stratified reporting, with lower engagement among minority ethnic participants and insufficient evidence to determine whether interventions effectively reduced ethnic disparities in gestational diabetes outcomes.

**Conclusions:**

Within the peer-reviewed evidence identified, interventions were delivered to multi-ethnic cohorts without documented cultural adaptation, and ethnicity-stratified reporting was too limited to permit an assessment of equity of effectiveness. These findings relate to the published literature and do not account for community-based provision not captured by this search. There is a clear need for co-designed, culturally responsive programmes evaluated in trials powered to detect ethnic differences in outcome.

## 1 Introduction

Maternal obesity is one of the most urgent public health issues of the 21st century, affecting millions of women and children around the world (2). According to the World Health Organization (WHO), 43% of women aged 18 years and above were overweight in 2025, compared with 25% in 1990 (3). This has a direct effect on pregnancy outcomes, as more women are becoming pregnant with a high body mass index (BMI) and face an increased risk of excessive gestational weight gain (GWG) beyond recommended levels (4). Among pregnant women with obesity and excess GWG, risks are elevated for gestational diabetes mellitus (GDM), pre-eclampsia, cesarean section, postpartum hemorrhage (OR 1.5, 95% CI 1.3–1.7), and venous thromboembolism (OR 2.5, 95% CI 2.0–3.2) (5). Maternal obesity is also strongly associated with large-for-gestational-age (LGA) babies (OR 2.2, 95% CI 2.0–2.4), birth injuries, and increased neonatal intensive care admissions (6). These risks extend into intergenerational cycles of obesity, as children born to obese mothers have a significantly higher predisposition to childhood and adult obesity (7).

This crisis is especially acute among minority ethnic groups in the United Kingdom. More than half of all pregnant women in England are overweight or obese (57%), with marked ethnic differences: 66.5% of Black women compared to 49% of White women enter pregnancy with a higher BMI (8). MBRRACE-UK data reveal stark disparities in maternal mortality: Black women face a risk of pregnancy-related death 3.9 times higher, and Asian women 1.8 times higher, than White women (9). Women of South Asian and Black Caribbean descent are three to five times more likely to develop GDM than White women (10).

These disparities are compounded by limitations in current risk stratification. Existing BMI thresholds do not adequately capture risk in minority ethnic groups. South Asian women, in particular, have higher visceral adiposity and metabolic dysfunction at lower BMI ranges, sometimes described as the thin-fat phenotype (11). Although NICE guidelines apply a reduced intervention threshold for South Asian women (23 kg/m^2^ vs. 25 kg/m^2^), many practitioners do not routinely apply ethnic-specific thresholds, leading to delayed identification and intervention (12). Independent evidence suggests that equivalent cardiometabolic risk may occur at BMI thresholds as low as 22.5 kg/m^2^ in some South Asian populations (12).

National guidance recommends weight management support for all pregnant women (NICE NG246; NICE NG247). Despite strong evidence of disproportionate burden, current interventions have consistently been found to be less effective at engaging minority ethnic women (13–15). Conventional, culturally non-adapted weight management approaches show substantially lower participation and retention rates among these groups. This is consistent with Leininger's Theory of Cultural Care, which argues that where interventions are delivered without regard to the cultural values, beliefs, and lifeways of recipients, both effectiveness and equity are unlikely (16).

Barriers to engagement operate at multiple levels. Nutritional guidelines developed around Western food patterns cannot readily be applied to traditional cuisines from diverse cultural contexts (17). Physical activity promotion strategies presuppose access to facilities and cultural acceptance of exercise in ways that do not reflect the lived realities of many minority ethnic women (18). Conventional healthcare delivery models do not adequately account for language barriers, experiences of discrimination, and time constraints that affect minority ethnic women disproportionately (19). Systematic reviews suggest that culturally sensitive interventions are two to four times more effective than generic programmes among minority ethnic populations (20, 21), yet such approaches are rarely implemented in maternal weight management.

This represents a critical gap between what the evidence shows works in practice and the availability of culturally appropriate solutions, sometimes described as the know-do gap (22). The existing evidence base is fragmented, and no single synthesis has examined interventions targeting pregnant minority ethnic women specifically within the UK setting. This scoping review aims to fill that gap by mapping the published evidence on interventions, exploring cultural adaptation processes, understanding engagement patterns across different ethnic communities, and identifying priorities for future research and practice.

## 2 Methods

### 2.1 Study design and framework

This scoping review followed the five-stage methodological framework proposed by Arksey and O'Malley (1), with refinements recommended by Levac et al. (23). Reporting adhered to the PRISMA Extension for Scoping Reviews (PRISMA-ScR) guidelines (24). The scoping review methodology was selected as the most appropriate approach for mapping heterogeneous literature involving different study designs, ethnic groups, and intervention strategies, and for identifying key concepts and gaps in this emerging field (25). The framework includes an optional fifth stage involving consultation with stakeholders. Given the MSc dissertation context, a formal stakeholder consultation exercise was not conducted; however, the review team includes clinical and public health expertise that informed the interpretation of findings, and the review question was developed in response to an identified gap in clinical and policy need.

### 2.2 Research question

Using the Population-Concept-Context (PCC) framework recommended for scoping reviews (25), the following research question was developed: “What evidence exists on interventions to support healthy weight management for pregnant women from minority ethnic groups in the United Kingdom?”

### 2.3 Eligibility criteria

#### 2.3.1 Population

Pregnant women from minority ethnic groups in the UK, including South Asian (Indian, Pakistani, Bangladeshi), Black (African, Caribbean, Black British), East Asian (Chinese, Japanese, Korean), Middle Eastern, mixed ethnic heritage, and other non-White groups as defined by the UK Office for National Statistics (26).

#### 2.3.2 Concept

Interventions aimed at supporting appropriate gestational weight gain or maintenance, promoting healthy lifestyle behaviors, and reducing the risk of obesity-related complications, including GDM. This encompassed dietary, physical activity, behavioral, educational, digital (mHealth), and multi-component approaches. In the UK, pregnant women are not routinely weighed throughout pregnancy; gestational weight management was therefore operationalised to include interventions targeting dietary behavior, physical activity, and related health outcomes in pregnant women, regardless of whether weight itself was the primary measured outcome.

#### 2.3.3 Context

UK healthcare and community settings, including National Health Service facilities (primary, secondary, and tertiary care), community centers, and remote or digital care delivery platforms.

#### 2.3.4 Exclusion criteria

Studies were excluded if they focused solely on a White British or European population; lacked ethnic disaggregation of data; did not have weight management, dietary behavior, or physical activity as a primary focus (studies in which weight or lifestyle was mentioned only incidentally within general antenatal care were therefore excluded); addressed clinical eating disorders; were not based in the United Kingdom; involved animal models; or were systematic reviews, literature reviews, editorials, or commentaries. Gray literature was also excluded, as set out in Section 3.4.

### 2.4 Search strategy and data sources

A search strategy was developed in collaboration with an evidence synthesis specialist and pilot-tested for sensitivity and specificity (25). Seven electronic databases were systematically searched from January 2010 to January 2025: MEDLINE, CINAHL, PsycINFO, PubMed, Scopus, Web of Science, and Global Health. The search period was selected to capture evidence generated following the publication of NICE PH27 guidance on weight management in pregnancy, which set the policy context for UK interventions. The search strategy combined medical subject headings (MeSH), subject headings, major headings, and keywords appropriate to each database (see Supplementary File 1). A top-up search was not conducted for the period 2025–2026; this represents a limitation acknowledged in Section 4.3.

### 2.5 Study selection

The database search returned 8,730 records. Following removal of 1,376 duplicates, 7,354 records were screened at the title and abstract stage. Of these, 7,032 were excluded as clearly irrelevant, leaving 322 for full-text review. A further 317 were excluded at full-text stage, with reasons reported in Figure 1. Five studies were provisionally retained. During data extraction, two of these [Poston et al. (29), the main UPBEAT trial report; and Simpson et al. (30), a cluster RCT of postnatal group weight management] were found to report no outcome or engagement data disaggregated by ethnicity, despite recruiting multi-ethnic cohorts (26% and 10.5% minority ethnic participants, respectively). They were therefore excluded against the pre-defined criterion requiring ethnic disaggregation, leaving three included studies.

**Figure 1:**
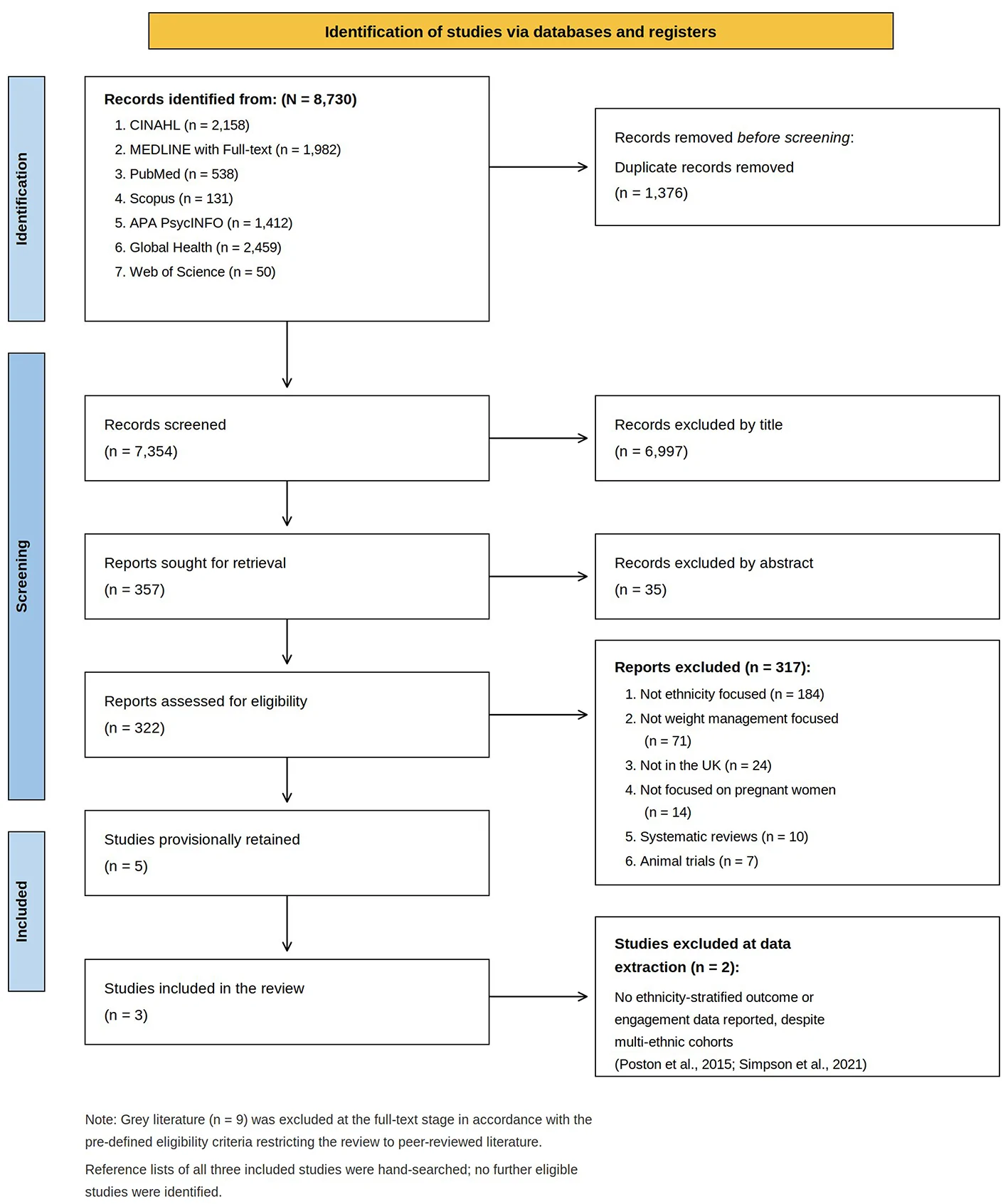
PRISMA-ScR flow diagram.

### 2.6 Study selection and data extraction

Records retrieved from all seven databases were exported to Rayyan systematic review software (27) for deduplication and screening. Titles and abstracts were screened against the pre-defined eligibility criteria by two reviewers (BM and SR), with discrepancies resolved by a third screener (BPV). Records retained at this stage underwent full-text review against the same criteria. As an additional source, the reference lists of all three included studies were hand-searched. The decision to restrict the review to peer-reviewed literature is addressed in Section 5.3. Record counts at each stage and the reasons for exclusion are reported in the esults (Section 4.1) and in the PRISMA-ScR flow diagram (Figure 1).

Data extraction was conducted using a standardized form capturing: study characteristics (author, year, location, design, sample size); population characteristics (ethnic composition, inclusion criteria); intervention characteristics (type, theoretical framework, cultural adaptations, delivery mode, setting, duration, intensity, provider); outcome characteristics (participation rates, retention, outcome measures); and barriers and facilitators to implementation and engagement.

### 2.7 Quality appraisal

As a scoping review following PRISMA-ScR guidelines, formal quality appraisal using a risk-of-bias tool is not a standard component of this methodology (25). However, several methodological considerations are worth noting. Flynn et al. (31) conducted a secondary analysis of dietary data from the UPBEAT RCT, with ethnicity-related findings derived *post-hoc*. Kusinski et al. (32) was a double-blind RCT with pre-specified secondary analyses, but ethnicity subgroup analyses were *post-hoc* and underpowered. Khunti et al. (33) included a pre-specified subgroup analysis by ethnicity for the primary outcome, representing the strongest methodological approach to ethnic disaggregation among the included studies, though the analysis remained underpowered. The limited number of included studies (*n* = 3) and the heterogeneity of their designs, populations, and outcomes precluded any formal meta-analysis or pooled effect estimation.

### 2.8 Data analysis and synthesis

Narrative synthesis was used to integrate findings across included studies (28). Three domains of interest were identified inductively through iterative review of study characteristics, intervention elements, and reported outcomes by two reviewers (BM and BPV), focusing on cultural adaptation processes, ethnic differences in engagement, and patterns of ethnicity-stratified outcome reporting. Disagreements were resolved through discussion and, where needed, a third reviewer (NA) was consulted. Quantitative data on participation and outcomes were tabulated to enable comparison across studies and ethnic groups.

## 3 Results

### 3.1 Characteristics of included studies

Three studies were included, published between 2016 and 2025, covering a total of 1,741 participants across ethnically diverse urban UK settings. Table 1 summarizes the key characteristics and findings of all included studies. Three domains were identified across the synthesis: multi-component behavioral interventions applied without cultural adaptation; differential engagement by ethnicity; and limited ethnicity-stratified reporting of effectiveness.

**Table 1:** Characteristics and key findings of included studies.

Author (Reference)	Study aim	Design	Setting	Sample/ Ethnicity	Intervention	Key findings (ethnicity-relevant)
Flynn et al. (31)	Examine dietary patterns in obese pregnant women and associations with GDM risk.	Quantitative (RCT secondary analysis).	8 UK antenatal clinics (London, Bradford, Glasgow, Manchester, Newcastle, Sunderland).	*n* = 1,023; 64% White, 23% Black, 8% Asian, 5% Other.	Dietary and physical activity counseling targeting glycaemic load and saturated fat (UPBEAT trial).	Intervention reduced Processed and Snacks dietary pattern scores across the whole cohort (both *p* < 0.0001); no significant change in the African/Caribbean or Fruit and Vegetables patterns. The African/Caribbean pattern was associated with increased GDM risk across the whole cohort, independent of ethnicity (OR 2.46, 95% CI 1.41–4.30). No ethnicity-stratified engagement data reported.
Kusinski et al. (32) [DiGest]	Assess whether a reduced-energy diet during pregnancy improves maternal weight and neonatal outcomes in women with GDM.	Quantitative (double-blind RCT).	8 hospital centers, England.	*n* = 425; 78% White, 17% Asian, 4% Black.	Weekly diet boxes: 1,200 kcal/day (intervention) vs. 2,000 kcal/day (control); 40% carbohydrate, 25% protein, 35% fat.	No significant difference between-group difference in the primary outcomes (maternal weight change; birth weight). Intervention reduced long-acting insulin requirement (OR 0.36, *p* = 0.003). No significant ethnicity-by-intervention interaction for any outcome (all *p*-interaction >0.05), though non-White subgroups were small.
Khunti et al. (33) [Baby Steps]	Assess effectiveness of a hybrid group education and mHealth intervention to promote physical activity in women with prior GDM.	Quantitative (RCT).	Two NHS hospital sites in Leicester and Nuneaton.	*n* = 293; 60% White European, 34% South Asian, 6% Other.	Two group education sessions plus mobile web application (Baby Steps); co-produced with multi-ethnic patient and stakeholder groups.	No significant between-group difference in physical activity (0.95 mg, *p* = 0.185). Intervention improved self-efficacy, anxiety, and quality of life. Engagement lower among minority ethnic participants: group attendance White 89% vs. minority ethnic 71%; app registration White 75% vs. minority ethnic 48%.

### 3.2 Multi-component behavioral interventions applied without cultural adaptation

Each of the three included studies delivered multi-component lifestyle interventions to cohorts that included minority ethnic participants. None documented a systematic cultural adaptation process in programme design or delivery.

Flynn et al. (31) used factor analysis of dietary data from 1,023 UPBEAT trial participants to identify four dietary patterns: Fruit and Vegetables, African/Caribbean, Processed, and Snacks. The intervention significantly reduced Processed pattern scores (adjusted difference −0.14, 95% CI −0.19 to −0.08, *p* < 0.0001) and Snacks pattern scores (−0.24, 95% CI −0.31 to −0.17, *p* < 0.0001) at 28 weeks, with effects maintained at 36 weeks. There was no significant change in the African/Caribbean pattern at either time point. The African/Caribbean pattern was associated with an increased risk of GDM across the whole cohort, independent of ethnicity (OR 2.46, 95% CI 1.41–4.30), though it was most prevalent among Black participants. The reduction in Processed and Snacks pattern scores was observed across the whole cohort and was not reported separately by ethnicity. The African/Caribbean pattern, characterized by foods such as plantain, cassava, and rice, is typically higher in carbohydrate, and Flynn et al. (31) did not report why the intervention did not alter it. One plausible explanation is that the dietary advice, which targeted glycaemic load and saturated fat reduction without engaging with the composition of traditional African and Caribbean diets, was less applicable to women following those diets. The association between this pattern and GDM was adjusted for maternal age, body mass index, and parity, but residual confounding cannot be excluded.

In the DiGest trial (32), 425 women with GDM and a BMI of 25 kg/m^2^ or above were recruited across eight English hospital centers (78% White, 17% Asian, 4% Black). Participants received either a 1,200 kcal/day diet box (intervention) or a 2,000 kcal/day diet box (control), both with the same macronutrient composition (40% carbohydrate, 25% protein, 35% fat). No significant differences between groups were found on the primary outcomes of maternal weight change to 36 weeks (adjusted difference −0.20 kg, 95% CI −1.01 to 0.61, *p* = 0.63) or standardized birth weight. The intervention group required significantly less long-acting insulin (OR 0.36, 95% CI 0.18–0.70, *p* = 0.003); this effect was not reported separately by ethnic group. Pre-prepared diet boxes were provided to all participants regardless of cultural background, without culturally specific dietary options.

The Baby Steps trial (33) was the only included study with a deliberate multi-ethnic recruitment target and documented co-production involvement of multi-ethnic patient and stakeholder groups. The reported co-production involved consultation with patient and stakeholder groups during an iterative design, testing and refinement pathway; the published account does not specify the depth of minority ethnic involvement in decision-making, nor whether the resulting content was adapted for specific cultural or linguistic groups. Of 293 women with a history of GDM recruited across two NHS hospital sites in Leicester and Nuneaton, 60% were White European, 34% South Asian, and 6% from other ethnic groups. Participants were randomized to a hybrid intervention of two group education sessions delivered 2 weeks apart, based on the Let's Prevent Diabetes programme (34) and targeting a daily increase of 30 min of moderate physical activity, plus access to a mobile web application, or to routine care. All intervention participants received a wrist-worn activity tracker. The primary outcome of objectively measured physical activity at 12 months did not differ significantly between groups (adjusted difference 0.95 mg in daily average acceleration, approximately equivalent to 500 steps per day, 95% CI −0.46 to 2.37, *p* = 0.185). Significant improvements were found in secondary outcomes including exercise self-efficacy (adjusted difference 0.54, 95% CI 0.05–1.02, *p* = 0.029), anxiety (−0.91, 95% CI −1.74 to −0.09, *p* = 0.031), and quality of life (0.05, 95% CI 0.004–0.09, *p* = 0.032).

### 3.3 Differential engagement by ethnicity

Khunti et al. (33) reported engagement data explicitly by ethnic group. Attendance at group education sessions was 89% among White European participants and 71% among those from minority ethnic backgrounds. The disparity was larger for the digital component: 75% of White European women registered for the mobile web application compared with 48% of minority ethnic women. Overall, 83% of intervention participants attended at least one session and 77% attended both, but these aggregate figures obscured the ethnic difference. App engagement was limited across both groups: 30% viewed monthly booster sessions and 30% used the chat forum, with no ethnicity-specific breakdown reported for these features. Khunti et al. (33) identified lower engagement among minority ethnic participants as a key finding and recommended that future trials develop strategies to improve acceptance and long-term adherence in high-risk minority ethnic groups. Digital literacy, language accessibility of the app interface, and the cultural relevance of the content were cited as likely contributors to the registration gap.

None of the three studies reported study attrition or withdrawal disaggregated by ethnicity. DiGest reported similar overall withdrawal rates between trial arms (13.7% and 14.0%), and Baby Steps reported 12-month retention, but neither examined whether loss to follow-up differed by ethnic group. This is itself an instance of the reporting gap this review documents: had attrition been higher among minority ethnic participants, as the engagement data might suggest, it would further limit what can be concluded about effectiveness in these groups. Flynn et al. (31) did not report attendance by ethnicity but noted that lower educational attainment, associated with minority ethnic status in the UPBEAT sample, was linked to lower scores on the Fruit and Vegetables dietary pattern, a pattern generally taken to indicate poorer overall diet quality.

### 3.4 Limited ethnicity-stratified reporting of effectiveness

Across all three included studies, ethnicity-disaggregated outcome data were reported either in an exploratory, underpowered, or implicit manner rather than in a pre-specified and adequately powered analysis. Across these three studies, this limits what can be established about equity of effectiveness.

The DiGest trial (32) made the most concerted attempt to disaggregate outcomes by ethnicity. *Post-hoc* subgroup analyses examined whether the intervention's effect on long-acting insulin requirement, time in range, large-for-gestational-age births, and postnatal HbA1c varied by ethnic group. No statistically significant ethnicity-by-intervention interaction was found for any outcome, but the authors noted that non-White subgroup sizes were small, and these analyses lacked the statistical power to confirm the absence of differential effects.

In Baby Steps (33), a pre-specified subgroup analysis of the primary physical activity outcome by ethnicity found no significant interaction. The authors acknowledged that within-group sample sizes were insufficient to detect differential treatment effects with confidence.

The most substantive ethnicity-related finding came from Flynn et al. (31), who identified the African/Caribbean dietary pattern and its distinct association with GDM risk. The observation that an increment in baseline African/Caribbean pattern scores carried an adjusted odds ratio for GDM of 2.46 (95% CI 1.41–4.30), combined with the finding that the intervention did not change this pattern, represents an implicit but important ethnicity-relevant result. The interaction between ethnicity and the intervention's effect on dietary patterns was not formally tested, and the paper did not report whether the failure to alter the African/Caribbean pattern was consistent across Black participants or varied by level of dietary acculturation.

Taken together, within these three studies it is not possible to determine whether any reviewed intervention was more or less effective for minority ethnic women than for White women, because the effectiveness data were not reported in a way that allows ethnic groups to be compared.

## 4 Discussion

### 4.1 Summary of main findings

Three UK studies published over approximately 10 years constitute the peer-reviewed evidence identified by this search. Importantly, none of the three was designed specifically for minority ethnic pregnant women; each was conducted in a multi-ethnic cohort and reported some ethnicity-disaggregated data. From 8,730 screened records, only three met the inclusion criteria. This scarcity reflects both the restrictive eligibility criteria applied here, particularly the requirement for ethnicity-disaggregated reporting, and the exclusion of gray literature, which limits the comprehensiveness of this evidence map. The requirement for ethnicity-disaggregated reporting is likely to be the principal reason so few studies qualified: interventions were often delivered to multi-ethnic cohorts, but their outcomes were not reported in a way that allowed ethnic groups to be distinguished. This aligns with the argument of Nazroo et al. (35) that health research continues to produce evidence without mandated data disaggregation, and so perpetuates the production of findings presented as universal but that cannot speak to whether interventions work for minority ethnic groups.

Available interventions were implemented with multi-ethnic cohorts but without meaningful cultural adaptation. Resnicow et al. (36) drew a useful distinction between surface structure adaptation, which involves including minority ethnic individuals in a programme, and deep structure adaptation, which involves redesigning the programme to fit the cultural values, food practices, and social contexts of those individuals. All three studies achieved the former at best. The failure of the UPBEAT programme to alter the African/Caribbean dietary pattern (31), while reducing Processed and Snacks pattern scores across the wider cohort, illustrates the clinical cost of surface-level inclusion. The African/Caribbean pattern was associated with GDM risk across the whole cohort, independent of ethnicity, and was the dietary pattern least reached by the intervention. Whether a culturally adapted dietary component would have changed this cannot be answered from the available data, but the gap is unsurprising given that UPBEAT targeted glycaemic load and saturated fat reduction without engaging with the cultural composition of participants' diets. Kumanyika (37) argues that generic dietary advice fails because it does not account for the lived environment and symbolic meaning of traditional foods. The same observation applies to DiGest (32), where pre-prepared diet boxes with standardized macronutrient ratios were provided to all participants regardless of cultural background.

The engagement gap provides the most direct evidence of systemic failure. The 18 and 27 percentage-point differences in group attendance and app registration between White and minority ethnic participants in the Baby Steps trial (33) are not trivial. Taylor et al. (38) found that structural barriers, particularly time, childcare, and transport, affected women from marginalized communities disproportionately. Importantly, both Taylor et al. (38) and Khunti et al. (33) found that minority ethnic women were motivated to participate; the barriers were practical and structural, not motivational. Addressing those barriers requires more than flexible scheduling. It involves locating programmes within community settings that minority ethnic women already use, adopting peer support models that reduce the power distance of clinical interactions, and evaluating digital components for accessibility and cultural appropriateness before implementation rather than discovering engagement disparities *post-hoc*.

The routine absence of ethnicity-stratified outcome data carries the most direct policy implications. Equitable services cannot be commissioned without disaggregated effectiveness data, because equity cannot be assessed from aggregate results alone. The DiGest subgroup analyses were exploratory and underpowered. The UPBEAT trial enrolled 26% Black participants but reported no ethnicity-stratified primary outcomes. This reporting pattern is not a technical constraint. It reflects decisions about which questions are treated as mandatory and which as optional in trial design, decisions that are themselves shaped by institutional norms about whose outcomes require separate scrutiny. Nazroo et al. (35) characterize this as institutional racism operating through routine research practice rather than intent. A related tension concerns whether interventions should be culturally tailored to specific groups or designed around cultural humility as a general principle; the studies reviewed here adopted neither approach explicitly, and this question requires dedicated research. NICE (12) highlights that minority ethnic women face elevated cardiometabolic risks at lower BMI thresholds than White women, yet without stratified outcome data, the effectiveness of interventions at those thresholds remains unknown. The absence of ethnicity-stratified reporting is not a uniquely UK problem: reviews from the United States, Canada, and Australia similarly report that ethnicity-stratified outcomes are seldom published in maternal weight management research, which situates the UK gap within a wider international pattern of inadequate data disaggregation.

### 4.2 Implications for practice and policy

For clinicians, the evidence reviewed here provides no assurance that referring minority ethnic pregnant women to standard weight management provision will produce outcomes equivalent to those seen in White women, because none of the included studies reported effectiveness in a way that allows this comparison to be made. Midwives and all healthcare professionals involved in antenatal care need both time and cultural competency to engage meaningfully with the dietary situations, family contexts, and structural pressures of the women they care for. Routine antenatal contacts are often too short for that kind of interaction, and 68% of midwives surveyed by McCann et al. (39) reported low confidence in their ability to address weight with minority ethnic women. Investment in training, not simply reassurance, is needed to address this. Ethics committees and research funders also have a role to play in reversing the norm of underpowered ethnicity subgroup analyses, by making pre-specified ethnic stratification a condition of funding for trials in diverse populations.

For commissioners, scaling up existing programmes is unlikely to produce equitable outcomes unless those programmes were designed with equity as an explicit goal. New investment should be directed toward co-designed development processes in which minority ethnic communities are genuine participants in intervention design rather than recruitment targets. Follow-up analyses must be structured to enable ethnic subgroup analysis from the outset. Community and third sector organizations, including voluntary, community and social enterprise (VCSE) partners already delivering culturally responsive provision alongside the NHS, should be treated as partners in design and delivery rather than as referral routes. In the interim, while new evidence is awaited, existing programmes can be modified in ways that address the structural barriers identified here: community venue delivery, flexible scheduling, interpreter provision, and evaluation of digital components for accessibility before rollout.

### 4.3 Strengths and limitations

The breadth of the database search, covering 8,730 records across seven databases, is a strength of this review and exceeds the scope of comparable searches in the existing literature. Adherence to the Arksey and O'Malley (1) framework and PRISMA-ScR reporting guidelines ensures transparency and reproducibility. A range of intervention types was reviewed, from large RCTs to community-based and digital programmes, providing an overview of the current UK activity. The decision to report only on what primary studies stated specifically about minority ethnic participants, rather than drawing inferences from aggregate data, consciously limits interpretive overreach.

Several limitations should be acknowledged. Three included studies are insufficient for generalization, and this low yield warrants reflection. The principal driver is the combination of eligibility criteria applied: UK setting, English language, peer-reviewed publication, and ethnicity-disaggregated reporting. The low yield is consistent with a genuine gap in the ethnicity-disaggregated evidence rather than search insensitivity alone, given the breadth of the search and the number of iterations conducted (Supplementary File 1). The exclusion of two large UK trials at the data extraction stage (29, 30) is itself illustrative: both recruited substantial multi-ethnic cohorts, including 26% and 10.5% minority ethnic participants respectively, yet neither reported any outcome disaggregated by ethnicity. Their exclusion reflects a reporting gap in the primary literature rather than an absence of relevant intervention research. Broadening the geographic scope to other high-income countries, or including studies without ethnicity-disaggregated data, would have increased the number of records but could not have answered the equity question this review poses. The review was deliberately restricted to peer-reviewed, English-language literature in order to map the formally published evidence base against a consistent standard of reporting and methodological transparency. This decision carries a clear consequence: NHS service evaluations, community programme reports, and VCSE-led initiatives are seldom published in peer-reviewed journals, and relevant provision of this kind will not have been captured. The evidence base mapped here should therefore be read as the peer-reviewed literature rather than the full range of UK activity, and this is a genuine limitation of scope rather than one the review can claim to have overcome. The reference lists of all three included studies were hand-searched as a supplementary step, though this is not a substitute for a systematic search of gray literature and yielded no further eligible studies. A top-up search to cover 2025–2026 was not conducted. The COVID-19 pandemic disrupted both maternity service delivery and research recruitment during part of the search window, which may have affected the volume and nature of evidence generated. NHS England policy initiatives, including the Maternity Transformation Programme (40), represent relevant activity that a peer-reviewed search of this design would not capture. All three studies used broad ethnic categories that obscure important within-group cultural and religious variation: women of Pakistani and Bangladeshi origin have distinct dietary practices and healthcare experiences compared to Indian women, and women of Nigerian background differ substantially from those of Jamaican heritage. A scoping review can only absorb that limitation from its primary studies, and the broad Asian and Black categories used in the available literature cannot generate the granular knowledge needed to develop culturally specific interventions.

### 4.4 Conclusions

Pregnant women from minority ethnic groups in the UK carry a disproportionate burden of obesity-related complications. Within the peer-reviewed evidence identified here, the interventions studied were delivered to multi-ethnic cohorts without documented cultural adaptation. Where engagement was reported by ethnicity, it was substantially lower among minority ethnic women. The routine absence of ethnicity-stratified outcome reporting means that equity of effectiveness cannot currently be assessed from this literature. These observations apply to the peer-reviewed studies identified; they do not account for community-based and VCSE-led provision that this search was not designed to capture, and which existing evidence suggests may be more culturally responsive. Given the disproportionate burden of obesity-related complications carried by minority ethnic pregnant women, future research must be built around co-designed interventions developed with the communities they are intended to serve, and adequately powered trials with pre-specified, and ideally mandated, ethnic subgroup analyses. In parallel, healthcare services must recognize the limitations of the current evidence base for populations that have been historically underserved in research, and act to ensure equitable, culturally appropriate access to weight management care during pregnancy. Applying generic programmes to high-risk populations and describing that as equitable care is not sufficient, and it has real consequences for the women it affects.

## Data Availability

The original contributions presented in the study are included in the article/Supplementary material, further inquiries can be directed to the corresponding author.
