# Supplementary material for: Weight management interventions for pregnant women from minority ethnic populations living in the United Kingdom: a scoping review
Source: Front Public Health. 2026 Sep 25;14:1885429. doi: 10.3389/fpubh.2026.1885429 (PMC13649400; doi:10.3389/fpubh.2026.1885429)
Supplement: Supplementary file 1 [file Table_1.docx]

**Supplementary File 1: Search Strategy**

*Weight Management Interventions for Pregnant Women from Minority Ethnic Populations Living in the United Kingdom: A Scoping Review*

Manuscript ID: 1885429 | Frontiers in Public Health

This file reports the search strategy for all seven electronic databases searched between January 2010 and January 2025. The strategy was structured around the Population–Concept–Context (PCC) framework and combined free-text keywords with database-specific controlled vocabulary (subject headings or index terms). Searches were limited to English-language, peer-reviewed publications.

The strategy was developed iteratively. For each database, an initial broad search was piloted and then progressively refined to improve specificity while retaining sensitivity to the review question. The number of iterations varied by database, from one run in PubMed to eight in MEDLINE. Section 2 reports the yield of every run; Section 3 gives the full final search string for each database.

**1. Search Concepts**

Four concept blocks were combined with the Boolean operator AND, with terms within each block combined using OR. The following terms formed the basis of the strategy across all databases, with controlled vocabulary adapted to each platform.

| **PCC element** | **Free-text terms** |
| --- | --- |
| **Population** | "Pregnancy" OR "Pregnant" OR "maternal" OR "antenatal" OR "ante-natal" OR "perinatal" OR "peri-natal" OR "gestation" AND "Ethnic minority" OR "BAME" OR "BME" OR "ethnic group" OR "multicultural" OR "multi-cultural" OR "multiethnic" OR "multi-ethnic" OR "South Asian" OR "Indian" OR "Pakistani" OR "Bangladeshi" OR "Black African" OR "Black Caribbean" OR "African" OR "Caribbean" OR "Chinese" OR "migrant" OR "immigrant" |
| **Concept** | "Healthy Weight Management" OR "weight manage" OR "weight gain" OR "gestational weight gain" OR "GWG" OR "obesity" OR "obese" OR "overweight" OR "body mass index" OR "BMI" OR "healthy weight" OR "diet" OR "dietary" OR "nutrition" OR "physical activity" OR "exercise" OR "sedentary" OR "behaviour change" OR "behavioural" OR "lifestyle" OR "intervention" OR "support" OR "counselling" OR "educat" OR "health promotion" OR "program" OR "programme" |
| **Context** | "United Kingdom" OR "UK" OR "Great Britain" OR "Britain" OR "British" OR "England" OR "English" OR "Scotland" OR "Scottish" OR "Wales" OR "Welsh" OR "Northern Ireland" OR "NHS" OR "National Health Service" |

**2. Iterative Search Development**

The table below records every search run conducted in each database and its yield. The final run for each database, shown in bold, produced the record count reported in Figure 1.

| **Database** | **Run** | **Approach** | **Records** |
| --- | --- | --- | --- |
| **MEDLINE (EBSCOhost)** | 1 | Keywords and index terms combined, 2010–2025 | 115,719 |
|  | 2 | Keywords only | 16,135 |
|  | 3 | Index terms only | 39,798 |
|  | 4 | Reduced term set | 82,882 |
|  | 5 | Further reduced term set | 61,756 |
|  | 6 | Further reduced term set | 29,544 |
|  | 7 | Further reduced term set | 4,333 |
|  | **8 (final)** | Fewest terms; most specific | **1,982** |
| **CINAHL (EBSCOhost)** | 1 | Keywords and index terms combined | 5,456 |
|  | 2 | Index terms only | 1,614 |
|  | 3 | Keywords only | 2,757 |
|  | 4 | Reduced term set | 4,454 |
|  | 5 | Further reduced term set | 2,541 |
|  | **6 (final)** | Fewest terms; most specific | **2,158** |
| **APA PsycINFO (EBSCOhost)** | 1 | Keywords and index terms combined | 8,647 |
|  | 2 | Keywords only | 5,911 |
|  | 3 | Index terms only | 2,411 |
|  | 4 | Reduced term set | 4,541 |
|  | **5 (final)** | Fewest terms; most specific | **1,412** |
| **Global Health (EBSCOhost)** | 1 | Keywords and index terms combined | 5,208 |
|  | 2 | Keywords only | 2,237 |
|  | 3 | Index terms only | 2,024 |
|  | **4 (final)** | Reduced term set | **2,457** |
| **PubMed** | **1 (final)** | Keywords and MeSH terms combined | **538** |
| **Scopus** | 1 | Full keyword set | 20 |
|  | **2 (final)** | Reduced keyword set | **131** |
| **Web of Science** | 1 | Full keyword set | 18,358 |
|  | 2 | Reduced keyword set | 4,974 |
|  | **3 (final)** | Fewest terms; most specific | **2,341** |

*Note: The Global Health and Web of Science yields recorded during searching (2,457 and 2,341 respectively) differ slightly from the figures reported in Figure 1 (2,459 and 50). These differences are documented here for transparency; the Figure 1 counts reflect the records exported to Rayyan following the application of the peer-review and full-text limiters at the point of export.*

**3. Final Search Strings**

The following are the complete search strings for the final run in each database, reproduced as executed.

**3.1 MEDLINE with Full Text (EBSCOhost) — Run 8, 1,982 records**

( "Pregnancy" OR "Pregnant" OR (MH "Pregnancy+") AND "Ethnic minority" OR "ethnic group" (MH "Ethnicity+") OR (MM "Ethnic and Racial Minorities") OR (MH "Asian People+") OR (MH "African People+") OR (MM "Asian") ) AND ( “Weight management interventions” OR “weight management programmes” OR "Healthy Weight Management" OR "Healthy weight" OR "intervention" OR "Health promotion" OR (MH "Body Weight Changes+") OR (MH "Weight Gain+") OR (MH "Obesity Management+") OR (MM "Pregnancy in Obesity") OR (MH "Health Promotion+") ) AND ( "United Kingdom" OR "UK" "Great Britain" OR "Britain" OR "British" "England" OR "English" "Scotland" OR "Scottish" "Wales" OR "Welsh" "Northern Ireland" OR (MH "United Kingdom+") OR (MH "England+") OR (MH "Scotland+") OR (MH "Wales") OR (MM "Northern Ireland") OR (MM "Ireland") )

**3.2 CINAHL Complete (EBSCOhost) — Run 6, 2,158 records**

( "Pregnancy" OR "Pregnant" OR "maternal" OR "antenatal" AND "Ethnic minority" OR “Asian” OR "African" OR "Caribbean" OR (MH "Pregnancy+") OR (MM "Obesity, Maternal") AND "Ethnic minority" OR “Asian” OR "African" OR "Caribbean" OR (MM "Linguistic Minorities") OR (MH "Ethnic Groups+") OR (MH "Minority Groups+") OR (MH "Asians+") OR (MH "Middle Eastern Persons+") OR (MH "Africans+") OR (MH "Caribbean Persons+") ) AND ( “Weight management interventions” OR “weight management programmes” OR "Healthy Weight Management" OR "Weight gain" OR "obese" OR "diet" OR "dietary" OR "nutrition" OR "Physical activity" OR "exercise" OR "intervention" OR "Health promotion" OR (MH "Weight Gain+") OR (MM "Weight Control") OR (MM "Weight Lifting") OR (MH "Obesity+") OR (MM "Obesity, Maternal") OR (MH "Diet+") OR (MH "Nutrition+") OR (MH "Exercise+") OR (MM "Physical Activity") OR (MH "Health Promotion+") OR (MM "Weight Reduction Programs") ) AND ( "United Kingdom" OR "UK" "Great Britain" OR "Britain" OR "British" "England" OR "English" "Scotland" OR "Scottish" "Wales" OR "Welsh" "Northern Ireland" OR (MH "United Kingdom+") OR (MM "England") OR (MH "Great Britain+") OR (MH "Scottish Persons") OR (MM "Scotland") OR (MM "Wales") OR (MM "Northern Ireland") )

**3.3 APA PsycINFO (EBSCOhost) — Run 5, 1,412 records**

"Pregnancy" OR "Pregnant" OR "antenatal" OR "maternal"OR DE "Pregnancy" OR DE "Adolescent Pregnancy" OR DE "Gestational Diabetes" OR DE "Preeclampsia" OR DE "Pregnancy" OR DE "Gestational Diabetes "Abortion" AND"Ethnic minority" OR "ethnic group"OR "African" OR "Caribbean" OR “Asian”OR DE "Racial and Ethnic Groups" OR DE "African Cultural Groups" OR DE "Asians" OR DE "Black People" OR DE "European Cultural Groups" OR DE "Indigenous Populations" OR DE "Latinos/Latinas" OR DE "Middle Eastern and North African Cultural Groups" OR DE "Multiracial" OR DE "Oceanian Cultural Groups" OR DE "People of Color" OR DE "South American Cultural Groups" OR DE "Central Asian Cultural Groups" OR DE "Chinese Cultural Groups" OR DE "Japanese Cultural Groups" OR DE "Japanese Americans" OR DE "Korean Cultural Groups" OR DE "South Asian Cultural Groups" OR DE "Southeast Asian Cultural Groups" OR DE "Vietnamese Cultural Groups" OR DE "American Indians" OR DE "Alaska Natives" OR DE "Inuit" OR DE "Indigenous Peoples in Australia" OR DE "Indigenous Peoples in Canada" OR DE "Pacific Islanders" OR DE "Hawaii Natives" OR DE "Mexican Americans" OR DE "Arab Cultural Groups" OR DE "Caribbean Cultural Groups" OR DE "Central American Cultural Groups" OR DE "Anglos" OR DE "Race and Ethnic Discrimination" OR DE "Cultural Sensitivity" OR DE "Multiculturalism" OR DE "Social Equity" OR DE "Minority Groups" OR DE "Sexual Minority Groups" AND “Weight management interventions” OR “weight management programmes” OR "Healthy Weight Management" OR"Healthy weight"OR "intervention"OR "Health promotion" OR "dietary" OR"Physical activity" OR "exercise"OR DE "Behavioral Neuroscience" OR DE "Diets" OR DE "Vegan Diet" OR DE "Vegetarian Diet" OR DE "Weight Control" OR DE "Healthy Eating" OR DE "Behavior" OR DE "Body Weight Cycling" OR DE "Behavior Modification" OR DE "Behavior Therapy" OR DE "Nutrition" OR DE "Calories" OR DE "Dietary Supplements" OR DE "Food" OR DE "Mealtimes" OR DE "Sugars" OR DE "Glucose" OR DE "Blood Sugar" OR DE "Fast Food" OR DE "Food Additives" OR DE "Food Insecurity" OR DE "Food Preparation" OR DE "Food Safety" OR DE "Eating Behavior" OR DE "Binge Eating" OR DE "Chewing" OR DE "Dietary Restraint" OR DE "Emotional Eating" OR DE "Food Refusal" OR DE "Health Behavior" OR DE "Exercise Dependence" OR DE "Health Risk Behavior" OR DE "Preventive Health Behavior" OR DE "Self-Care" OR DE "Food Intake" OR DE "Exercise" OR DE "Aerobic Exercise" OR DE "Weightlifting" OR DE "Yoga" OR DE "Obesity (Attitudes Toward)" OR DE "Body Mass Index" OR DE "Physique" OR DE "Weight-Based DiscriminationOR DE "Intervention" OR DE "Crisis Intervention" OR DE "Early Intervention" OR DE "Family Intervention" OR DE "Group Intervention" OR DE "Workplace Intervention" OR DE "Debriefing (Psychological)" OR DE "Parenting Interventions" OR DE "Parent Training" OR DE "Parent Educational Background" OR DE "Parental Attitudes" OR DE "Parental Expectations" OR DE "Parental Health" OR DE "Parental Mental Health" OR DE "Parental Occupation" OR DE "Parental Sensitivity" OR DE "Parental Stress" OR DE "Single Parents" OR DE "Stepparents" OR DE "Workplace Health Promotion" OR DE "Prevention" OR DE "Preventive Health Behavior" OR DE "Preventive Health Services" OR DE "Health Promotion" OR DE "Public Health Campaigns" AND "United Kingdom" OR "UK""Great Britain" OR "Britain" OR "British""England" OR "English""Scotland" OR "Scottish""Wales" OR "Welsh""Northern Ireland""NHS" OR "National Health Service"

**3.4 Global Health (EBSCOhost) — Run 4, 2,457 records**

"Pregnancy" OR "Pregnant" OR "maternal"OR "antenatal"OR DE "pregnancy" OR DE "pregnant women" OR DE "maternal nutrition" OR DE "maternal behaviour" OR DE "gestational diabetes" AND "Ethnic minority" OR “Asian” OR "African" OR "Caribbean" OR DE "ethnic groups" OR DE "African-Caribbeans" OR DE "Asians" OR DE "black people" OR DE "Hispanics" OR DE "white people" DE "black people" OR DE "Asia" OR DE "Central Asia" OR DE "East Asia" OR DE "South Asia" OR DE "South East Asia" OR DE "West Asia" OR DE "Africa" OR DE "Africa South of Sahara" OR DE "Anglophone Africa" OR DE "Francophone Africa" OR DE "North Africa" AND “Weight management interventions” OR “weight management programmes” OR "Healthy Weight Management" OR "Weight gain"OR "obese"OR "diet" OR "dietary" OR "nutrition" OR"Physical activity" OR "exercise" OR"intervention" OR"Health promotion" OR DE "community health services" OR DE "maternity services" OR DE "public health services" OR DE "primary health care" OR DE "weight reduction" OR DE "weight" OR DE "body weight" OR DE "overweight" OR DE "underweight" OR DE "obesity" OR DE "body mass index" OR DE "diet" OR DE "diets" OR DE "physical activity" OR DE "exercise" OR DE "feeding behaviour" OR DE "dieting" OR DE "behavioural changes" OR DE "intervention" OR DE "nutritional intervention" OR DE "health promotion" OR DE "Nutrition Education and Training program" AND "United Kingdom" OR "UK""Great Britain" OR "Britain" OR "British""England" OR "English""Scotland" OR "Scottish""Wales" OR "Welsh""Northern Ireland""NHS" OR "National Health Service"OR DE "UK" OR DE "Channel Islands" OR DE "Great Britain" OR DE "Isle of Man" OR DE "Northern Ireland" OR DE "Scotland" OR DE "Eastern Scotland" OR DE "Northern Scotland" OR DE "Scottish Highlands and Islands" OR DE "West Scotland" OR DE "England" OR DE "East Midlands of England" OR DE "Eastern England" OR DE "Northern England" OR DE "South East England" OR DE "South West England" OR DE "West Midlands of England"

**3.5 PubMed — Run 1, 538 records**

(("Pregnancy" OR "Pregnant" OR "maternal" OR "antenatal" OR "ante-natal" OR "gestation Pregnancy" OR "gestation" OR "Pregnancy"[Mesh]) OR "Mothers"[Mesh] OR "Maternal Nutritional Physiological Phenomena"[Mesh] OR "Maternal Welfare"[Mesh] OR "Maternal Health Services"[Mesh] OR "Maternal Deprivation"[Mesh] OR "Maternal Behavior"[Mesh] OR "Prenatal Education"[Mesh] OR "Prenatal Care"[Mesh] AND "Ethnic minority" OR "BAME" OR "BME" OR "ethnic group" OR "multicultural" OR "multi-cultural" OR "multiethnic" OR "multi-ethnic" OR "South Asian" OR "Indian" OR "Pakistani" OR "Bangladeshi "OR "Black African" OR "Black Caribbean" OR "African" OR "Caribbean" OR "Chinese" OR "Minority Groups"[Mesh] OR "Ethnic and Racial Minorities"[Mesh] OR "Black People"[Mesh] OR "Medicine, African Traditional"[Mesh] OR "West African People"[Mesh] OR "Sub-Saharan African People"[Mesh] OR "North African People"[Mesh] OR "Central African People"[Mesh] OR "East African People"[Mesh] OR "African People"[Mesh] OR "Caribbean Region"[Mesh] OR "Caribbean People"[Mesh]OR "Asian People"[Mesh]) AND ("Healthy Weight Management" OR "Weight gain" OR "Gestational weight gain" OR "GWG" "obesity" OR "obese" OR "overweight" OR "Body mass index" OR "BMI" OR "Healthy weight" OR "diet" OR "dietary" OR "nutrition" OR "Physical activity" OR "exercise" OR "Behaviour change" OR "behavioural" OR "lifestyle" OR "intervention" OR "support" OR "counselling" OR "counselling" OR "Health promotion" OR "programme" OR "Body Weight"[Mesh] OR "Weights and Measures"[Mesh] OR "Weight Reduction Programs"[Mesh] OR "Ideal Body Weight"[Mesh] OR "Weight Loss"[Mesh] OR "Weight-Bearing"[Mesh] OR "Weight Gain"[Mesh] OR "Weight Lifting"[Mesh] OR "Body Weight Changes"[Mesh] OR "Obesity"[Mesh] OR "Obesity, Abdominal"[Mesh] OR "Obesity, Morbid"[Mesh] OR "Pregnancy in Obesity"[Mesh] OR "Obesity Management"[Mesh] OR "Body Mass Index"[Mesh] OR "Overweight"[Mesh] OR "Diet"[Mesh] OR "Diet Therapy"[Mesh] OR "Diet Surveys"[Mesh] OR "Nutrition Therapy"[Mesh] OR "Nutrition Policy"[Mesh] OR "Parenteral Nutrition, Home"[Mesh] OR "Parenteral Nutrition, Home Total"[Mesh] OR "Nutrition Assessment"[Mesh] OR "Exercise"[Mesh] OR "Behavioral Symptoms"[Majr] OR "Life Style"[Mesh] OR "Healthy Lifestyle"[Mesh] OR "Methods"[Mesh] OR "Early Intervention, Educational"[Mesh] OR "Early Medical Intervention"[Mesh] OR "Crisis Intervention"[Mesh] OR "Psychosocial Intervention"[Mesh] OR "Social Work"[Mesh] OR "Health Promotion"[Mesh] OR "Resistance Training"[Mesh] OR "Government Programs"[Mesh])) AND ("United Kingdom" OR "UK" "Great Britain" OR "Britain" OR "British" "England" OR "English" "Scotland" OR "Scottish" "Wales" OR "Welsh" "Northern Ireland" "NHS" OR "National Health Service" OR "United Kingdom"[Majr])

**3.6 Scopus — Run 2, 131 records**

( ALL ( "Pregnancy" OR "Pregnant" OR "maternal" OR "antenatal" AND "Ethnic minority" OR "Asian" OR "African" OR "Caribbean" ) AND ALL ( "Weight management interventions" OR "weight management programmes" OR "Healthy Weight Management" OR "Weight gain" OR "obese" OR "diet" OR "dietary" OR "nutrition" OR "Physical activity" OR "exercise" OR "intervention" OR "Health promotion" ) AND ALL ( "United Kingdom" OR "UK" "Great Britain" OR "Britain" OR "British" "England" OR "English" "Scotland" OR "Scottish" "Wales" OR "Welsh" "Northern Ireland" "NHS" OR "National Health Service" ) ) AND PUBYEAR > 2009 AND PUBYEAR < 2026 AND ( LIMIT-TO ( LANGUAGE , "English" ) )

**3.7 Web of Science Core Collection — Run 3, 2,341 records**

The Web of Science searches were executed through the platform interface and saved as session URLs rather than as exported strings. The final run applied the following terms:

Population: "Pregnancy" OR "Pregnant" AND "Ethnic minority"

Concept: "Weight management interventions" OR "weight management programmes" OR "Healthy Weight Management" OR "Weight gain" OR "obese" OR "diet" OR "dietary" OR "nutrition" OR "Physical activity" OR "exercise" OR "intervention" OR "Health promotion"

Context: "United Kingdom" OR "UK" OR "Great Britain" OR "Britain" OR "British" OR "England" OR "English" OR "Scotland" OR "Scottish" OR "Wales" OR "Welsh" OR "Northern Ireland" OR "NHS" OR "National Health Service"

Timespan: 2010–2025

**4. Records Retrieved by Database**

| **Database** | **Records exported** | **Platform** |
| --- | --- | --- |
| CINAHL | 2,158 | EBSCOhost |
| Global Health | 2,459 | EBSCOhost |
| MEDLINE with Full Text | 1,982 | EBSCOhost |
| APA PsycINFO | 1,412 | EBSCOhost |
| PubMed | 538 | NCBI |
| Scopus | 131 | Elsevier |
| Web of Science | 50 | Clarivate |
| **Total records retrieved** | **8,730** | — |
| Duplicates removed | 1,376 | Rayyan |
| **Records screened at title and abstract** | **7,354** | Rayyan |

**5. Supplementary Searching**

The reference lists of all three included studies (Flynn et al., 2016; Khunti et al., 2023; Kusinski et al., 2025) were hand-searched. No further studies meeting the eligibility criteria were identified.

Grey literature, including NHS service evaluations, community programme reports and voluntary sector publications, was not searched. The rationale for restricting the review to peer-reviewed literature, and the implications of that decision, are addressed in Section 5.3 of the manuscript.

**6. Notes on Execution**

Field codes and controlled vocabulary tags differ across platforms and were adapted accordingly: (MH) and (MM) for CINAHL and MEDLINE subject headings on EBSCOhost; DE for APA PsycINFO and Global Health descriptors; [Mesh] and [Majr] for PubMed; ALL( ) for Scopus; and TS= for Web of Science.

Searches were executed by the first author (BM). The strategy was developed in consultation with the supervisory team and an evidence synthesis specialist, and was piloted for sensitivity and specificity before the final runs. All exported records were imported into Rayyan systematic review software for deduplication and screening.
